# Optimizing Binding Site Spacing in Fluidic Self-Assembly for Enhanced Microchip Integration Density

**DOI:** 10.3390/mi15030300

**Published:** 2024-02-22

**Authors:** Myeongho Park, Bin Yoo, Myeonghwan Hong, Daeun Cho, Yunjin Jeong, Cheolheon Park, Jaemin Kim, Tae-Min Ha, Garam Kim, Sang Jeen Hong, Daewon Lee

**Affiliations:** 1 Department of Electronics Engineering, Myongji University, Yongin 17058, Republic of Korea; mook4818@naver.com (M.P.); y090b090@gmail.com (B.Y.); audghks7245@naver.com (M.H.); ekdms1602@naver.com (D.C.); jaemin@esl.mju.ac.kr (J.K.); garamkim@mju.ac.kr (G.K.); 2 Semiconductor Equipment Engineering Program, Myongji University, Yongin 17058, Republic of Korea; samhong@mju.ac.kr; 3 Bio-MAX Institute, Seoul National University, Seoul 08826, Republic of Korea; yunjin.jeong.777@gmail.com (Y.J.); majamoolse@gmail.com (C.P.); 4 Department of Semiconductor Engineering, Myongji University, Yongin 17058, Republic of Korea; gertrude001@gmail.com

**Keywords:** scalable assembly, fluidic self-assembly, microchip, packaging, patterning process, optoelectronic devices, microLEDs, augmented reality (AR)

## Abstract

This manuscript presents a comprehensive study on the assembly of microchips using fluidic self-assembly (FSA) technology, with a focus on optimizing the spacing between binding sites to improve yield and assembly. Through a series of experiments, we explored the assembly of microchips on substrates with varying binding site spacings, revealing the impact of spacing on the rate of undesired chip assembly across multiple sites. Our findings indicate a significant reduction in incorrect assembly rates as the spacing increases beyond a critical threshold of 140 μm. This study delves into the mechanics of chip alignment within the fluid medium, hypothesizing that the extent of the alloy’s grip on the chips at different spacings influences assembly outcomes. By analyzing cases of undesired assembly, we identified the relationship between binding site spacing and the area of chip contact, demonstrating a decrease in the combined left and right areas of chips as the spacing increases. The results highlight a critical spacing threshold, which, when optimized, could significantly enhance the efficiency and precision of microchip assembly processes using FSA technology. This research contributes to the field of microcomponent assembly, offering insights into achieving higher integration densities and precision in applications, such as microLED displays and augmented reality (AR) devices.

## 1. Introduction

Pick-and-place transfer technology is a packaging technique that uses a robot machine to place different devices at specific locations on a circuit board [1]. Because of its excellent positioning accuracy, pick-and-place technology has been regarded as the most promising transfer technology in the packaging field, where a large number of micro-scale components need to be assembled. However, pick-and-place technology is a serial process and requires sophisticated equipment, and assembly speed and cost constraints are present [2,3]. Therefore, it is difficult to use this method in an application where tens of millions of micro-sized chips must be placed on a single substrate in the field of microLED display manufacturing [4,5,6,7]. In particular, as efforts are ongoing to achieve the high-density integration of micro-sized devices on limited circuit boards to achieve high performance, fluidic self-assembly transfer technology is attracting attention as a technology to complement this.

Fluidic self-assembly (FSA) technology allows for components to naturally interact and assemble within the fluid to form a desired structure by using the movement of fluid [6,7,8,9,10,11,12,13,14,15,16,17,18,19,20]. An irreversible bond is formed between a particle and an assembled substrate coated with molten alloy when a component comes into contact with one of the binding sites. This allows for the assembly of multiple particles in a scalable and massively parallel manner in a matter of seconds. This shows that FSA can not only form sophisticated structures and patterns faster and easier than the pick-and-place transfer method but also solve the time and equipment problems caused by the high-density integration of micro-sized devices by transferring a large number of components in a short time at a low cost. The pick-and-place transfer method can accurately assemble the positions of the components and substrates to be assembled one-to-one through mechanical control. However, because microdevices move randomly in the fluid and come into contact with designated locations during the FSA transfer process, it is important to guarantee accuracy and consistency in the assembly process. This means that depending on the device’s size or shape, it may be challenging to fabricate the desired structure by self-assembly if the assembly interaction between microcomponents and binding sites is not smooth. Therefore, in order to alleviate assembly difficulty considering the assembly characteristics of FSA, the gap between binding sites on the substrate must be made wider than the size of the components to be assembled to provide a margin. This margin allows for microcomponents to achieve precise assembly without interfering with each other through random movement. In the end, assembly through FSA technology has the limitation of low integration because it is difficult to keep the spacing between components very close.

However, the alignment through the existing FSA poses a limitation in terms of the integration density due to the difficulty in placing particles in close proximity to each other. For applications in devices like augmented reality (AR), there is a need for higher Pixels Per Inch (PPI) and reduced spacing between chips, necessitating denser assembly. Simply narrowing the gap between binding sites on the substrate leads to a problem where a single chip may incorrectly attach to two alloy binding sites simultaneously, resulting in undesired assembly. Once a chip is wrongly assembled and fixed in this manner, correcting it becomes exceedingly difficult and is in fact more problematic than a non-attachment scenario. In cases of non-assembly, alternative methods can be employed for further assembly, but when chips are mistakenly attached in an undesired manner, detaching these fine particles is extremely challenging. We discovered and analyzed a phenomenon, where reducing the gap between binding sites to a certain extent significantly increases the likelihood of a single chip attaching to two binding sites. We believe this is due to the chip achieving sufficient stabilization by contacting a sufficiently large area of the alloy on both sides.

In this study, we conducted experiments to analyze the situation where a single chip attaches in an undesired manner to two binding sites while adjusting the gap between these sites during the FSA process to bring the particles closer together and increase the integration density. The 3 × 2 cm^2^ PCB substrates were fabricated, and the size of the binding site, where the microcomponents are aligned, was designed to be 180 × 180 μm^2^. Si microchips, which play the role of microcomponents, were fabricated with a size of 220 × 220 × 80 μm^3^, and FSA experiments were conducted by reducing the spacing between the binding sites to various sizes. Through this experiment, we discovered that as the spacing between binding sites is reduced, two or more binding sites for one microcomponent become tangled together, reducing the assembly yield of the component. We present the size and tendency of the binding site gap that can increase integration by minimizing the gap between components while causing the phenomenon to occur the least.

## 2. Experimental Results and Discussions

**Figure 1:**
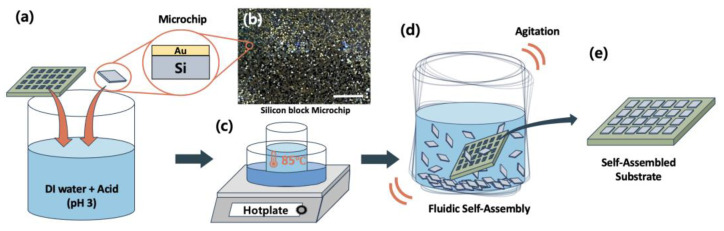
Overall schematic of the chip assemble process using FSA. (**a**) Assembly preparation process using an alloy-wetted substrate and chip. (**b**) Image of the microchip (220 μm); scale bars, 1 cm. (**c**) For the FSA process, the temperature of the vial containing the microchip and substrate was raised to 85 °C on a hotplate. (**d**) Schematic diagram of the FSA process. When the inside of the vial reaches approximately 85 °C, agitate it for assembly. (**e**) Schematic diagram of the final substrate with the chip alignment completed through FSA.

Arranging micro semiconductor components on a substrate with high density not only reduces the interconnection length between components, leading to decreased signal loss and improved reliability, but also results in reduced power consumption. In this way, achieving high integration also provides great advantages in applications that require the integration of many chips in a limited space, such as microLED displays, AR, optoelectronic devices [7], and mobile devices. Because the degree of integration of a substrate is determined by the number of components compared to the area, it is important to increase the number of components within a certain area. By reducing the binding site spacing, which is the distance between components on a PCB of the same area, more components can be arranged. If FSA technology is used for component assembly, it takes less than 1 min for thousands of components to be completely assembled [6,13,20]. In this respect, FSA is a way to list more components quickly and easily. FSA technology utilizes a phenomenon in which components stick to the alloy at temperatures above the melting point of the alloy arranged on the substrate [21]. After putting in a large amount of chips in the fluid and heating them, the substrate to be assembled is put in the fluid and agitated. As the chips move freely in the fluid, the surface of the alloy of the substrate is melted by the heated fluid, and it aligns itself when it touches the Au of the chip. In this experiment, as shown in Figure 1a, many 220 μm Si chips (Figure 1b) and Au deposited on a 3 × 2 cm^2^ alloy-laminated substrate were placed in water at pH 3 to remove the surface oxide. Afterward, FSA was performed by agitating the chip and substrate in heated water, as shown in Figure 1c,d. As a result, it was confirmed that when the binding site spacing is the same as the chip size or is larger than the chip size and there is a margin during assembly, one chip per assembly site is assembled very well (Figure 1e). However, when the binding site spacing is reduced, it was discovered that one chip is stuck to two alloy pads at the same time, and we predicted that this phenomenon would increase as the binding site spacing continued to be reduced. Even when the binding site spacing was increased to 140 μm, a small amount of chips was assembled in an undesired condition, but when the binding site spacing was less than 130 μm, we could see a rapid increase in undesired assembly. This means that although there are assembly limitations when reaching a certain binding site spacing, it is possible to reduce the binding site spacing compared to the chip size.

**Figure 2:**
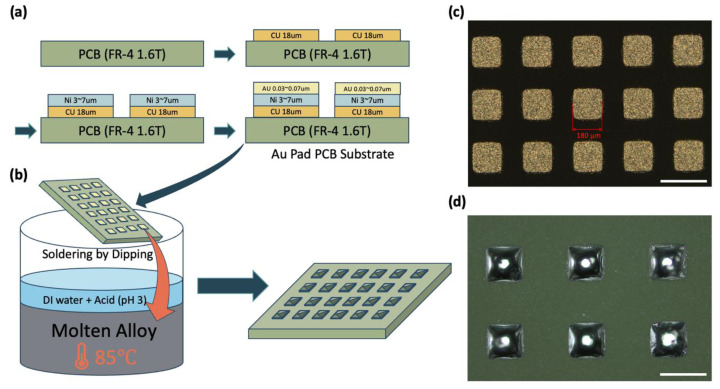
The process of manufacturing the necessary board and wetting solder on the board before proceeding with the FSA process. (**a**) They are etched to the desired pattern size after forming Cu, Ni, and Au on the FR-4 1.6T PCB substrate. (**b**) Dip in the dipping solution to form solder bumps. (**c**) The Au pattern, the wet layer where solder will be wetted, is listed; scale bars, 300 μm. (**d**) Solder bumps formed on the PCB board after process B; scale bars, 300 μm.

In analyzing the efficiency of FSA, we paid attention to changes in the binding site distance and used a PCB board for this purpose. In this experiment, an FR-4 1.6T PCB substrate with Cu, Ni, and Au formed sequentially was used, which is easy to mass produce and ensures product uniformity and reliability. (Figure 2a) The Au pattern regularly arranged on the PCB board is the core part where the alloy bump is formed by the alloy-wetting layer [21,22]. For this reason, layers of Cu and Ni were deposited on the PCB board to increase adhesion to the PCB board. The square Au pattern maintains equal spacing in all directions, which is suitable for research purposes. (Figure 2c) Alloy, a metallic material, is sensitive to oxide film, and this oxide film can interfere with wetting with Au on the surface of the alloy. Therefore, the surface oxide film on the solder solution was removed by immersing it in a dipping solution adjusted to pH 3 and melted alloy (Figure 2b), and the 62 degree melting point alloy was formed at the binding spot of the solder bump on the board [23]. (Figure 2d) The number of binding sites in all the substrates we designed is approximately 3000.

**Figure 3:**
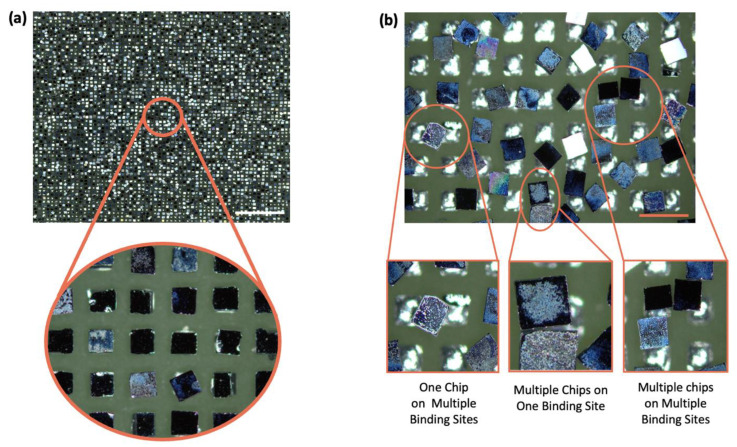
The FSA process is successfully completed, and the undesired assembly occurs. (**a**) A microscopic panoramic photograph of a normally completed FSA substrate; scale bars, 3 mm. (**b**) A panoramic photograph of a substrate with a 130 μm binding site spacing and three cases that affected the assembly; scale bars, 500 μm.

We predicted the occurrence of chips being assembled onto more than one binding site when the spacing between binding sites becomes smaller than the chip size. To validate this hypothesis, we fabricated PCB substrates in patterns with a binding site spacing less than 220 μm and conducted FSA to analyze the efficiency concerning different spacing. The substrates with spacing ranging from 120 μm to 160 μm were sequentially immersed in the fluid along with chips, and the assembly process was facilitated by agitation. As shown in Figure 3b, one chip attached to two or more binding sites, two chips attached to one binding site, and multiple chips adhered incorrectly to multiple binding sites, which are all examples of undesired multiple assemblies. The majority of the chips exhibit a case of undesired assembly, where a single chip spans across and attaches to two binding sites. These undesired assemblies are critical as they not only affect a single chip’s attachment to multiple binding sites but also have a cascading impact on nearby chips. As a result, if the tendency of parameters to influence this phenomenon can be investigated to rule out the effects of multiple assemblies, it can help increase the yield. In addition, reducing the spacing between the bonding sites without compromising the yield means increasing the area of the chip in the substrate and provides additional possibilities for improving the yield.

The PCB substrate with a binding site spacing of 140 μm or more exhibited an undesired assembly ratio below 4%, indicating a successful assembly shown in Figure 3a for the majority of chips. However, as the spacing narrowed, we observed a notable increase in the ratio of undesired assemblies, leading to a decline in the overall assembly yield. Parameters influencing this phenomenon include chip size, binding site size, and spacing. Among these, the binding site spacing exerted the most significant impact on the substrate area occupied by chips post-assembly. To investigate this undesired assembly, the chip size was fixed at 220 × 220 × 80 μm^3^, the binding site size was fixed at 180 × 180 μm^2^, and the binding site spacing varied from 120 μm to 160 μm, and the experiments were conducted using PCB substrates. As shown in Figure 3a, the substrate of 140 μm spacing demonstrated nearly exclusive one-chip assembly to one binding site. Conversely, on a 130 μm substrate, all three cases of multiple assembly occurred (Figure 3b), but the majority of instances involved a single chip attaching to two binding sites. These unforeseen occurrences during assembly disrupt the intended configurations and hinder the achievement of the desired assembly forms.

**Figure 4:**
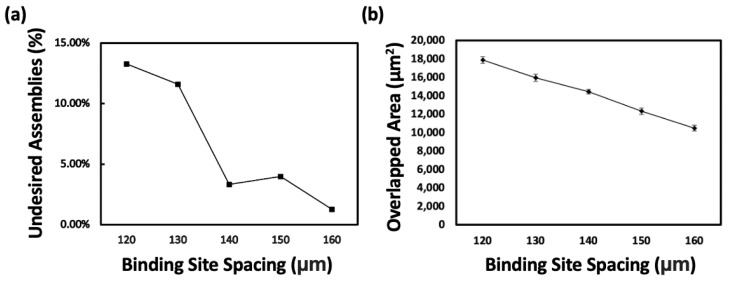
Graph trend according to undesired assemblies. (**a**) Graph of the incidence of undesired assemblies according to binding site spacing. (**b**) Graph of the total overlapped area of chip and binding sites according to binding site spacing.

We attempted to assemble microchips on substrates with five different binding site spacings using FSA technology. The yield of chips incorrectly assembles to two binding sites compared to the total number of binding sites, indicating undesired assembly, which can be depicted as shown in Figure 4a. As the spacing between binding sites increased from 120 μm to 160 μm in increments of 10 μm, the rates of incorrect assembly were 13.28%, 11.60%, 3.33%, 3.98%, and 1.28%, respectively. We observed a significant decrease in the rate of undesired assembly when the gap between the binding sites was above 140 μm. Once undesired assembly occurs, it becomes very difficult to repair and significantly impacts subsequent processes. We hypothesized that this phenomenon could depend on how strongly each alloy at the binding sites holds the chip when it encounters two binding sites while moving within the fluid. That is, when the distance between binding sites is short, the area of the chip held by each alloy would be larger, and conversely, when the distance is longer, the area held by each alloy would be smaller.

**Figure 5:**
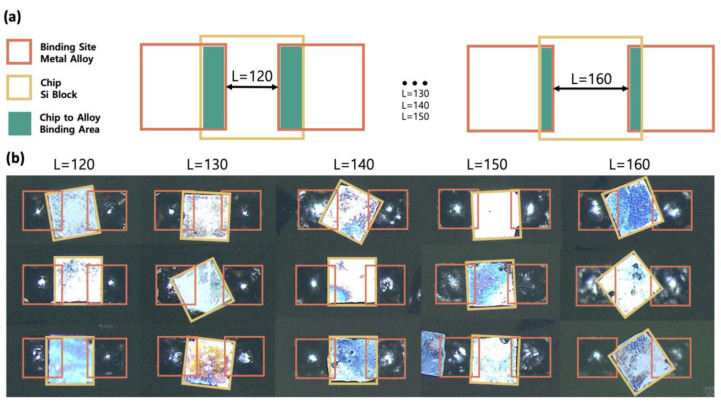
Analysis on undesirably assembled chips. (**a**) Conceptual diagram of how one chip (yellow) assembled on two binding sites (orange). (**b**) Binding site spacing ranging from 120 μm to 160 μm with 10 μm increments; actual chip and binding sites are overlayed by yellow and orange indicator.

Examples of further analysis on undesirably assembled chips are shown in Figure 5, where we analyzed how a chip is assembled between two binding sites and the left and right areas of the chip spanning across both binding sites. The average sum of the left and right areas can be seen in Figure 4b; the areas are 17,879, 15,969, 14,452, 12,334, and 10,493 μm^2^ with binding site spacings of 120, 130, 140, 150, and 160 μm, respectively. As expected, the sum of the left and right areas decreases as the physical gap between the binding sites increases, confirming a trend that matches the physical gap results. Through this experiment, we explored the correlation between the spacing of binding sites and its impact on undesired chip assembly. As a result, we identified the limit to minimizing the spacing of binding sites to reduce the likelihood of undesired chip assembly, thus enabling conditions for denser chip assembly.

## 3. Materials and Methods

### 3.1. Substrate Preparation

On the FR-4 1.6T PCB substrate, a Cu layer with a thickness of 18 μm and a Ni layer with a thickness of 3~7 μm were deposited sequentially as an adhesion layer. Afterward, the Au layer, which will be used as the alloy-wetting layer, was deposited at 0.03~0.07 μm. The substrate was designed with a pattern featuring pad sizes of 180 μm and a binding site spacing ranging from 120 μm to 160 μm with 10 μm increments. To prepare the dipping solution, alloy with melting point of 62 °C (In 51.29%, Sn 14.23%, and Bi 34.49%; by weight) were placed in a stainless steel pot. DI-water (50 mL) was added to the pot, and the alloy was completely melted by heating it to 85 °C. To lower the pH to 3 for the purpose of removing surface oxides on the molten solder, 100 μL of hydrochloric acid (HCl) was added to the dipping solution. Subsequently, flux was applied to remove the oxide layer on the Au pads of the substrate. After the complete melting of the solder, the substrate was immersed in the dipping solution for 10~15 s and agitated. The substrate was taken out of the stainless steel pot and then rinsed with ethanol and further washed with DI-water. Finally, moisture was eliminated using an air gun.

### 3.2. Chip Preparation

Before initiating the FSA process, we fabricated 220 × 220 μm^2^ chips. On a 4-inch Si wafer, 30 nm thick Ti and 300 nm thick Au were deposited with an adhesion layer. After grinding through backgrinding, the wafer was polished with the CMP process to produce a thickness of 80 μm. Later, we crafted 220 × 220 μm^2^ chips through dicing.

### 3.3. FSA Process

The assembly solution was prepared by adding hydrochloric acid (HCl) to DI-water to reduce the pH to 3. Approximately 220 × 220 μm^2^ chips were added to the vials filled with assembly solution, which was then heated to 85 °C using a hotplate. Next, substrates with solder bumps formed by alloy wetting were placed into the vials, which were then sealed with lids. The assembly underwent manual agitation for 10 s. Subsequently, the substrate was taken out of the vials and then rinsed with ethanol and further washed with DI-water. Finally, moisture was eliminated using an air gun.

## 4. Discussion

Our investigation into the optimization of binding site spacing via FSA technology offers novel insights into the microchip assembly process, emphasizing the intricate balance between spacing and assembly yield. The experimental results demonstrate a critical threshold in binding site spacing at 140 μm, above which the undesired assembly of the microchip is significantly decreased. The observed decrease in undesired assembly rates—from 13.28% at a 120 μm spacing to 1.28% at a 160 μm spacing, with substantial improvement observed beyond the 140 μm threshold—highlights the pivotal role of spacing in achieving high-precision assemblies. This finding is particularly relevant for producing high-density electronic devices, where the accurate placement of microcomponents is crucial. The relationship between binding site spacing and assembly yield underscores the need for precise control over the microfabrication process, suggesting that even minor adjustments in spacing can have profound effects on the overall assembly outcome. The implications of our study extend beyond the laboratory, offering tangible benefits for the fabrication of advanced electronic devices. By identifying the optimal binding site spacing for FSA, manufacturers can potentially improve the reliability and performance of devices, such as microLED displays and AR systems.

The critical spacing threshold, a pivotal factor in the microchip assembly yield and to avoid undesired assembly, is influenced by a constellation of variables including but not limited to the chip’s dimensions, the mass, and the fluid dynamics involved in the assembly process. The complexity of these factors necessitates a multidisciplinary approach to fully understand and predict the assembly behavior under varying conditions. Future research endeavors will focus on exploring the limits of spacing optimization further. The pursuit of a deeper theoretical understanding will aim to establish a robust model that accurately predicts the interactions and outcomes of the assembly process based on the aforementioned variables. This expanded research focus will address the gaps identified in the current study and respond to the call for a more detailed theoretical exploration. By doing so, we aim to provide a comprehensive framework that not only supports our empirical findings but also offers predictive insights that could guide the design and optimization of microchip assembly processes in many applications.

## 5. Conclusions

In conclusion, our investigation into the optimization of binding site spacing using FSA technology has revealed crucial insights into the assembly process of microchips. Our experiments have demonstrated that a critical threshold for binding site spacing exists, beyond which the rate of undesired assembly significantly diminishes, thereby enhancing the precision and yield of microchip assemblies. This finding is pivotal for the advancement of microcomponent assembly techniques, particularly in high-density applications where precision is paramount. By meticulously analyzing the undesired assembly phenomenon and its dependency on binding site spacing, we have provided a robust foundation for future research aimed at maximizing the efficiency of FSA processes. Ultimately, our study not only advances the understanding of FSA technology but also opens new avenues for its application in the fabrication of advanced electronic devices, offering a pathway toward higher integration densities and improved device performance in sectors ranging from microLED displays to AR technologies.

## Data Availability

The data that support the findings of this study are available on request from the correspondence author (D.L.) upon reasonable request.
